# Cold priming uncouples light- and cold-regulation of gene expression in *Arabidopsis thaliana*

**DOI:** 10.1186/s12870-020-02487-0

**Published:** 2020-06-18

**Authors:** Andras Bittner, Jörn van Buer, Margarete Baier

**Affiliations:** Plant Physiology, Freie Universität Berlin, Dahlem Centre of Plant Sciences, Königin-Luise-Straße 12-16, 14195 Berlin, Germany

**Keywords:** Cold, Light, Priming, Triggering, Memory, *Arabidopsis thaliana*, Growth, Defence, Gene expression

## Abstract

**Background:**

The majority of stress-sensitive genes responds to cold and high light in the same direction, if plants face the stresses for the first time. As shown recently for a small selection of genes of the core environmental stress response cluster, pre-treatment of *Arabidopsis thaliana* with a 24 h long 4 °C cold stimulus modifies cold regulation of gene expression for up to a week at 20 °C, although the primary cold effects are reverted within the first 24 h. Such memory-based regulation is called priming. Here, we analyse the effect of 24 h cold priming on cold regulation of gene expression on a transcriptome-wide scale and investigate if and how cold priming affects light regulation of gene expression.

**Results:**

Cold-priming affected cold and excess light regulation of a small subset of genes. In contrast to the strong gene co-regulation observed upon cold and light stress in non-primed plants, most priming-sensitive genes were regulated in a stressor-specific manner in cold-primed plant. Furthermore, almost as much genes were inversely regulated as co-regulated by a 24 h long 4 °C cold treatment and exposure to heat-filtered high light (800 μmol quanta m^− 2^ s^− 1^). Gene ontology enrichment analysis revealed that cold priming preferentially supports expression of genes involved in the defence against plant pathogens upon cold triggering. The regulation took place on the cost of the expression of genes involved in growth regulation and transport. On the contrary, cold priming resulted in stronger expression of genes regulating metabolism and development and weaker expression of defence genes in response to high light triggering. qPCR with independently cultivated and treated replicates confirmed the trends observed in the RNASeq guide experiment.

**Conclusion:**

A 24 h long priming cold stimulus activates a several days lasting stress memory that controls cold and light regulation of gene expression and adjusts growth and defence regulation in a stressor-specific manner.

## Background

Plants respond dynamically to a wide range of environmental signals and can adjust to many unfavourable conditions [1–3]. Performance optimization to persisting shifts is called acclimation or acclimatization. It takes several days and involves cost-intensive changes in metabolism, gene expression and sometimes even in the anatomy and morphology [4, 5]. Specific signalling, such as by the cold-induced ICE (inducer of CBF expression)-CBF (C-repeat binding factor)-pathway [6] and e.g. ROS (reactive oxygen species) and abscisic acid signalling conjointly configure the plants towards activation and manifestation of higher stress tolerance [1, 7]. As soon as the conditions improve, most acclimation supporting reactions stop almost immediately and reverting regulation starts [8–10].

If the lag-phases between successive stress events, which are by themselves too short to establish protection, are short enough to maintain part of the acclimation responses, several short stimuli can lead to similar or higher stress tolerance than a continuous stress experience [11]. The phenomenon is called entrainment.

By contrast, priming is independent of the persistence of the stress or of accumulation of primary stress responses [12]. The stress memory (caused by the priming stimulus) uses information carriers that are set at low metabolic costs and modify the response to a later stress (triggering stress) [12, 13]. Priming has been described for a wide range of biotic and abiotic stress stimuli [12–14]. However, in most cases the (precise) nature of the specific memory mechanism is still unknown. According to the first records, it can range from meta-stable metabolic imprints to trans-generation stable epigenetic marks [13, 15, 16].

In our earlier study on cold-priming, we showed that priming of *Arabidopsis thaliana* for 24 h at 4 °C differentially regulates genes of the core environmental stress response cluster, which are induced in response to various stressors, including cold [17, 18]. Cold priming weakened the induction of the zinc finger transcription factor *ZAT10* (*zinc-finger transcription factor 10*; *STZ*; At1g27730) (and to a lesser extent *BAP1* (*BON1-associated protein 1*; At3g61190)) upon a 5 day later cold stimulus and supported cold activation of *CHS* (*chalcone synthase*; *TT4*; At5g13930) and *PAL1* (*phenylalanine ammonium lyase 1*; At2g37040) expression [17]. The same priming stimulus did not affect cold-induction of *COR15A* (At2g42540) [17], which is under control of the main cold acclimation regulating ICE-CBF-pathway [1].

In this small selection of genes, *ZAT10* showed the strongest primability [17]. *ZAT10* expression responds to a wide range of abiotic stresses, including high light intensities and cold [17–19]. The transcription factor mediates secondary gene expression regulation, such as induction of the non-plastid ascorbate peroxidase *APX2* (At1g07890) and chloroplast iron superoxide dismutase *FSD1* (At4g25100) and counteracts full activation of osmotic and salt tolerance [20].

*ZAT10* is hardly expressed under non-stress conditions [17, 21]. In response to photooxidative stress, which occurs upon sudden cold or excess light [22–24], it is induced by reactive oxygen species (ROS), presumably by H_2_O_2_ [25]. In high light, *ZAT10* induction is supported by PAP (3′-phosphoadenosine 5′-phosphate) that accumulates upon photooxidative inhibition of the PAP-dephosphorylating chloroplast stroma localized phosphatase SAL1 (At5g63980) [26]. In the cold, CBF-dependent induction of the transcription factor CZF1 (At2g40140) activates *ZAT10* expression [27, 28]. The various *ZAT10* regulating pathways are differently controlled by chloroplast antioxidant protection. Whereas, for example, SAL1 regulation by ROS depends more on stromal ascorbate peroxidase (sAPX) function than on thylakoid ascorbate peroxidase (tAPX) activity [26], cold regulation of *CBF* genes is antagonized by tAPX [29] and cold priming of *ZAT10* is solely mediated by transient post-cold accumulation of tAPX and can be antagonized by *tAPX* RNA silencing [17, 30].

Cold mainly slows down enzymatic reactions, whereas excess light increases the excitation pressure in the photosynthetic light reaction with only low impact on energy consumption [22–24], Despite the different nature of both perturbations, cold and excess light cause both imbalances between photosynthetic electron transport and redox energy consuming chloroplast metabolism. Consistent with the high similarity of the effects on photosynthesis, the two stress types regulate 87% of the responsive genes in the same direction in naïve plants [31]. Many cold-responsive genes, e.g. *BAP1* and the ZAT (*Zinc finger of Arabidopsis thaliana*) transcription factors *ZAT6* (At5g04340), *ZAT10* and *ZAT12* (At5g59820) [20, 32–34] belong to the group of “core environmental stress response genes” that are induced in response to various stresses and mediate stress response regulation and acclimation processes [18]. The high overlap between transcriptome regulation in response to cold and light stress [31] suggests a strong *trans*-effect of cold-priming on light-regulation of gene expression. On the contrary, the complexity of regulation of primary stress responsive genes, like *ZAT10*, let assume *cis*- and *trans*-specific effects. In the present study, we compare the effect of 24-h cold priming on the response to a 5 day later applied 4 °C or temperature-controlled high light (800 μmol photons m^− 2^ s^− 1^) triggering stimulus, first, on frequently with *ZAT10* co-regulated genes and, finally, in a transcriptome wide scale to investigate the specificity of cold-priming on future gene expression regulation.

## Results

### Cold priming results in decreased cold activation of specific ZAT genes

**Fig. 1:**
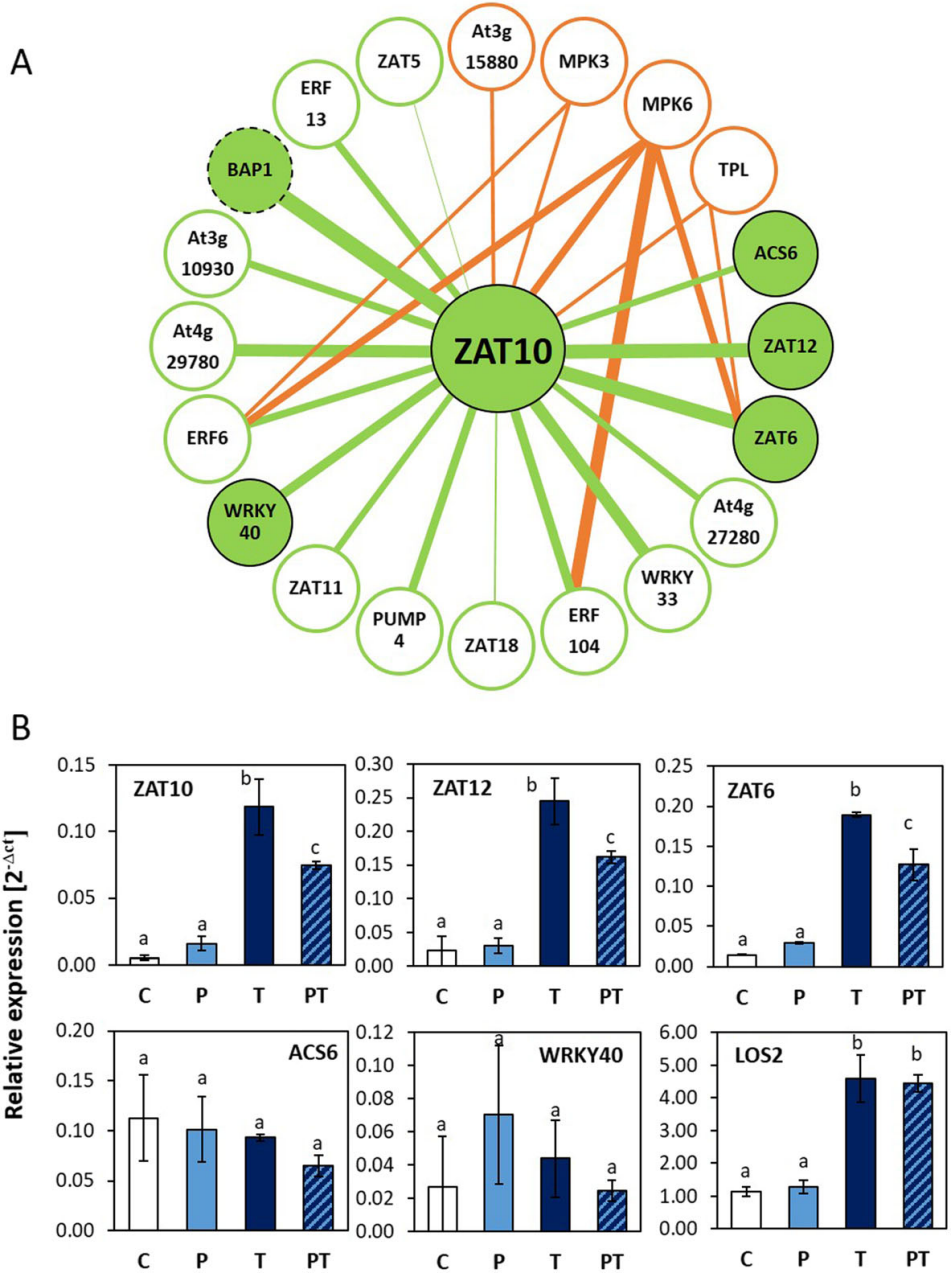
**a** Genes frequently coregulated with *ZAT10* (green) and proteins interacting with ZAT10 (orange) according to GENEMANIA and STRING. The thicker the connecting lines are drawn, the more studies reported co-regulation or interaction. Filled circles highlight the genes that were chosen for qPCR analysis. **b** Effect of 24 h cold priming at 4 °C on cold-regulation of 4 genes co-regulated with *ZAT10* in various studies and of the *ZAT10* upstream-regulator LOS2**.** Regulation of the relative transcript abundances (standardized on *YLS8*; mean ± standard deviation) in control plants (C), only cold-primed (P), only cold-triggered (T) and cold-primed + cold-triggered plants (PT) immediately after triggering. Different letters label statistical significance of differences based on data obtained with 3 independently cultivated and treated biological replicates (Tukeys post hoc test; *p* < 0.05)

*ZAT10* showed in the previous study strongest primability of the selected cold-responsive genes [17]. To identify similarly regulated genes in *Arabidopsis thaliana*, publicly available data resources on transcript abundance regulation were scanned with GENEMANIA for *ZAT10*-like regulated genes [35]. The 15 highlighted genes (Fig. 1a) included *BAP1*, which is, like *ZAT10*, cold-priming sensitive and less inducible by cold 5 days after 24 h cold priming at 4 °C, as shown before [17]. Additionally, GENEMANIA also named the genes for the zinc-finger transcription factors *ZAT6* (Zinc finger protein 6; At5g04340), *ZAT11* (At2g37430), *ZAT12* (At5g59820), *ZAT5* (At2g28200), *ZAT18* (At3g53600), the WRKY transcription factors *WRKY33* (At2g38470) and *WRKY40* (At1g80840), the AP2-type transcription factors *ERF6* (Ethylene response factor 6; At4g17490), *ERF13* (At2g44840) and *ERF104* (At5g61600), the mitochondrial uncoupling protein *PUMP4* (At4g24570) and the Ca^2+^-binding protein encoding gene At4g272800. A similar analysis on the STRING v.11 platform [36] showed also *ACS6* (1-aminocyclopropane-1-carboxylate synthase 6; At4g11280), that is involved in ethylene biosynthesis, as a *ZAT10* co-expressed gene (Fig. 1). All these genes respond, like *ZAT10*, to a wide range of abiotic stress stimuli and to oxidative stress [20, 37–43].

STRING v.11 further indicates protein-protein interactions (Fig. 1; orange lines). Via feed-back effects, they could impact on transcript abundance regulation. The ZAT10 transcription factor interacts with the MAP kinases MPK3 (At3g45640) and MPK6 (At2g43790), which are elements of a core plant stress signal transduction pathway responding to biotic and abiotic signals [44, 45]. MPK6 and MPK3 also phosphorylate ZAT6 [46], ERF6, ERF104 [41, 43], WRKY33 [47], WRKY40 [41] and ACS6 [48]. Additionally, ZAT10 interacts with the transcriptional co-repressors TOPLESS (TPL, At1g15750) and TOPLESS-RELATED-4 (At3g15880) [44, 49–51]. TPL binds also ZAT6 [50]. To test *ZAT10*-like regulated genes for the cold-primability of their cold regulation, we selected genes with different affinity to MPK6 / MPK3 and / or TPL, namely *ZAT10*, *ZAT6*, *ACS6* and *WRKY40* for a qPCR (quantitative polymerase chain reaction)-based priming analysis. We further included the gene for the bi-functional enolase *LOS2* (At2g36530), which is a negative upstream transcriptional regulator of *ZAT10* [52]. The transcript levels of these genes were analysed by qPCR immediately after triggering in previously naive plants (T) and in plants that were cold-primed 5 days before cold triggering (PT). As controls, untreated plants (C) and plants (P) that perceived 5 days earlier the priming cold-treatment, but were not cold-triggered, were analysed.

Like *ZAT10*, the transcript levels of *ZAT12* and *ZAT6* were significantly decreased in PT-plants as compared to T-plants, demonstrating priming-sensitivity (Fig. 1b). *ACS6* and *WRKY40* were not sensitive to the triggering stimulus, independent of whether the plants were cold-primed or not. Regulation of *LOS2*, which binds the *ZAT10* promoter and controls *ZAT10*-mediated cold-induction of the cold and drought marker gene *RD29* [52], was strongly cold-inducible (comparison of transcript levels in C- and T-plants), but not priming-regulated (comparison of transcript levels in T- and PT-plants). The analysis gave no indication that interaction with known ZAT10-interacting proteins controls priming, but demonstrated that cold-priming affects specific genes, even in a group of genes which are otherwise widely co-regulated with *ZAT10* [17–19] (Fig. 1).

### The effect of cold priming on the regulation of the ZAT genes upon high light triggering

**Fig. 2:**
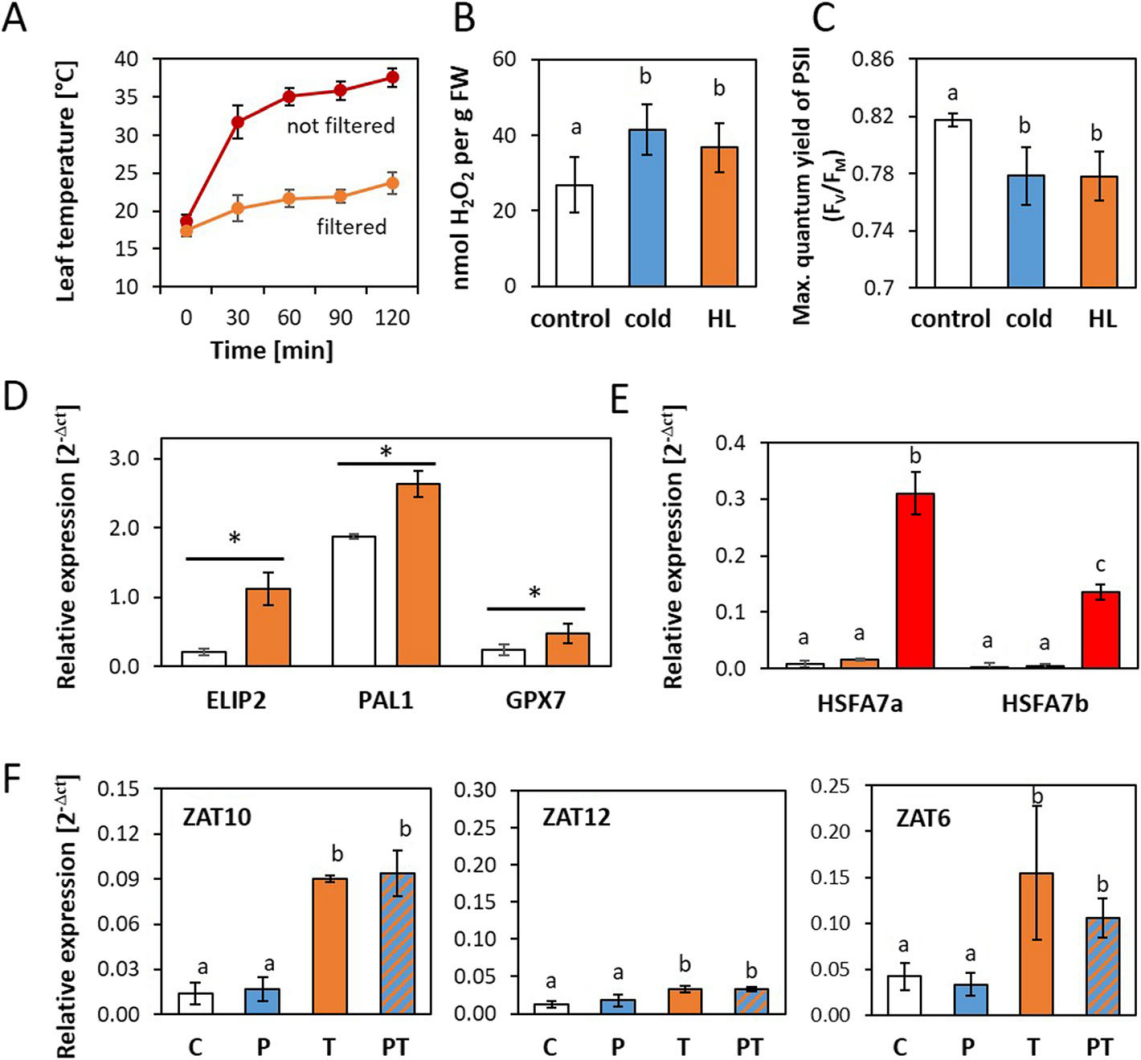
Effect of cold priming on light triggering. **a** Leaf surface temperature in the heat-filtered (orange) and in the not heat-filtered illumination set-up (red). **b** H_2_O_2_ content in control plants (white) and cold (blue) or high light triggered plants (orange). Different letters show statistical significance of differences based on data obtained with 9 plants from two independently cultivated and treated plant sets (Student t-test; *p* < 0.05). **c** Maximum quantum yield of photosystem II (F_V_/F_M_) in control plants (white) and cold (blue) or high light triggered plants (orange). The parameter was determined with a saturating white light flash after 20 min dark acclimation. Different letters show statistical significance of differences based on data obtained with 10 plants from two independently cultivated and treated plant sets (Student t-test; *p* < 0.05). **d** Relative transcript abundance of light-responsive genes in control plants (white) and after 2 h heat-controlled illumination (orange). The transcript levels were standardized on the transcript levels of *YLS8*; Statistically significant differences in the relative transcript abundances are labelled with asterisks (*n* = 3–5; Tukeys post hoc test; *p* < 0.05). **e** Relative transcript abundance of heat-responsive genes in control plants (white) and after 2 h heat-controlled illumination (orange) and not heat-controlled illumination (red). The transcript levels were standardized on the transcript levels of *YLS8*; Statistically significant differences in the relative transcript abundances are labelled with different letters (*n* = 3–5; Tukeys post hoc test; *p* < 0.05). **f** Effect of 24 h cold priming at 4 °C on transcript abundance regulation by a light stimulus. Regulation of the relative transcript abundances in control plants (C), only cold-primed (P), only light-triggered (T) and cold-primed + light-triggered plants (PT) immediately after light triggering. Different letters show statistical significance of differences in the relative transcript levels based on data obtained with 3–5 independently cultivated and treated biological replicates (Tukeys post hoc test; *p* < 0.05). All subfigures show means ± standard deviation

For comparison of the cold-priming effect on cold and high light triggering, we established a heat filtered high light set-up (800 μmol quanta m^− 2^ s^− 1^) (Fig. 2a), which increases H_2_O_2_ levels and damages photosystem II (as indicated by the maximum quantum yield of photosystem II) to a similar extent as the 4 °C treatment used for cold priming and cold triggering does (Fig. 2b and c).

The set-up was further evaluated by qPCR for its impact on regulation of well characterized light and heat regulated genes. After 2 h in high light, the transcript levels of the light-inducible genes *ELIP2* (*early light induced protein 2*, At4g14690 [53];), *GPX7* (*glutathione peroxidase 7*, At4g31870 [54];) and *PAL1* (*phenylalanine ammonium lyase 1*, At2g37040) were increased (Fig. 2d). The heat filter was sufficient to counteract significant activation of the heat sensitive genes *HSFA7a* (At3g51910) and *HSFA7b* (At3g63350) [55, 56] (Fig. 2e).

Besides induction of *ZAT10*, the light treatment increased the *ZAT6* transcript levels almost as strong as the 24 h cold treatment (T-plants in Figs. 1b and 2f). *ZAT12* showed only a very weak (but also significant) response to the light treatment (Fig. 2f). In cold-primed plants, the mean transcript levels of *ZAT6* were lower in PT-plants than in T-plants, indicating primability, although the effect was not significant due to strong variation of the gene induction level. On the contrary, the transcript levels of *ZAT10* and *ZAT12* were more similarly regulated by light triggering in primed and non-primed plants (Fig. 2f). Consequently, cold priming did not have any or had only very little effect on the light triggering response of these genes.

### Photosynthetic performance after triggering

**Fig. 3:**
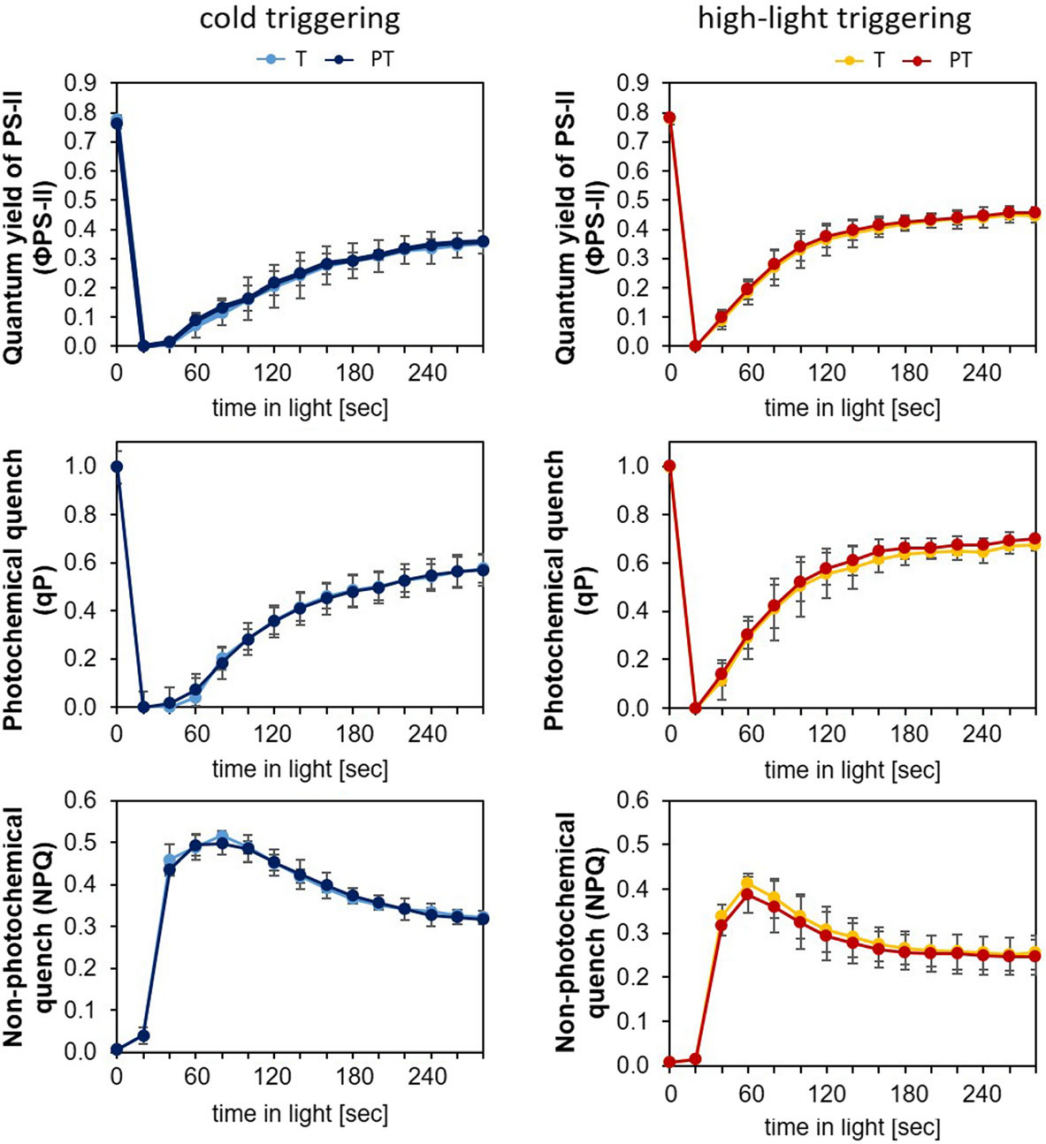
Effect of 24 h cold priming at 4 °C on photosynthetic electron transport activity and regulation after cold (left) and light triggering (right). The means and standard deviations of the quantum yields of photosystem II (Φ_PS-II_), photochemical quenching (qP) and non-photochemical quenching (NPQ) as determined for each of the 4 biological replicates at an photosynthetic photon flux density of 185 μmol quanta m^− 2^ s^− 1^ in parallel in triggered (T) and primed + triggered plants (PT)

The differences between the cold and the light triggering response of the *ZAT* genes in cold-primed plants (Figs. 1b and 2f), especially *ZAT10*, could result from effects of priming on the photosynthetic electron transport efficiency. To test this hypothesis, we compared the photosynthetic performance of photosystem-II in cold-primed plants after cold and light triggering by chlorophyll-a fluorescence analysis. Triggered (T) and primed + triggered (PT) plants were analysed side-by-side by 2-dimensional chlorophyll-a fluorescence imaging in middle-aged leaves, which show strongest priming sensitivity in 4-week-old plants [30] (Fig. 3).

After cold and light triggering, the maximal quantum yield of photosystem-II (F_V_/F_M_; 0 min in Fig. 3 top) was similar in dark-acclimated T- and PT-plants, demonstrating that the triggering responses were unaffected by cold priming. Upon illumination with a photosynthetic photon flux density (PPFD) of 185 μmol quanta m^− 2^ s^− 1^, the quantum yields of photosystem II (Φ_PS-II_) and photochemical and non-photochemical quenching (qP and NPQ) also did not differ between primed and non-primed plants, both, after cold- and after light-triggering (T- and PT). It demonstrated that the priming treatment did not reduce the response to the triggering stress, although cold and light by itself differently impacted on Φ_PS-II_ and the quenching parameters (Fig. 3). The similarity of the responses between the respective T- and PT-plants did not support the hypothesis that the priming-dependent differences in gene expression regulation result from differences in stress-induced damage or regulation of photosystem-II activity as caused by priming.

### Effect of cold priming on cold- and high light-regulated gene expression

For more insight into the effect of cold priming on the stress responses, we maximally widened the target gene spectrum and performed a genome wide RNA-sequencing (RNASeq analysis) experiment 2 h after cold (4 °C) and light triggering (800 μmol quanta m^− 2^ s^− 1^) of 5 days earlier cold-primed and non-primed plants. RNA sequencing resulted in 23.76–24.14 million reads per sample (Suppl. Tab. 1). At minimum, 98.49% of the reads could be mapped to the TAIR10 genome (Suppl. Tab. 1). Sequences were recorded for 24,085 different genes. The transcript levels of many well-known, highly cold and light-responsive transcription factors, e.g. *CBF1* (At4g25490) and *CBF3* (At4g25480) [57], *ANAC078* (At5g04410) [58] and *ZAT10* [17, 21] and *ZAT6* [59], were 2 h after cold or light triggering already strongly decreased (Suppl. Tab. 2). At the same time, the transcript levels of secondarily cold regulated genes, such as the CBF3-regulated gene *COR15A* (At2g42540) and the *ANAC078* target genes At1g56650, At3g01600 and At5g58610 [60] still were induced (Suppl. Tab. 2). Genes that are well characterized for their heat inducibility, such as *HSFA2* (At2g26150), *HSFA7a* (At3g51910), and *HSA32* (At4g21320), were only very weakly expressed in all samples (Suppl. Tab. 2). The transcript level of the senescence regulating NAC transcription factor *ORE1* (ANAC092; At5g39610) [61] was not increased in any sample (Suppl. Tab. 2). The expression pattern confirmed high responsiveness of stressor-specific target genes and showed that the treatments did not induce heat signalling or activate senescence.

61.7% of the genes that were at least 2-fold up-regulated and 32.8% of the genes at least 2-fold down-regulated in response to light in unprimed plants, were also at least 2-fold regulated by the cold treatment. On the contrary, only 0.3 and 5.5% of the at least 2-fold regulated genes were inversely regulated by cold and light. Thus, our cold and light treatments widely regulated genes in the same direction in unprimed plants, similar as shown before by others [31].

**Fig. 4:**
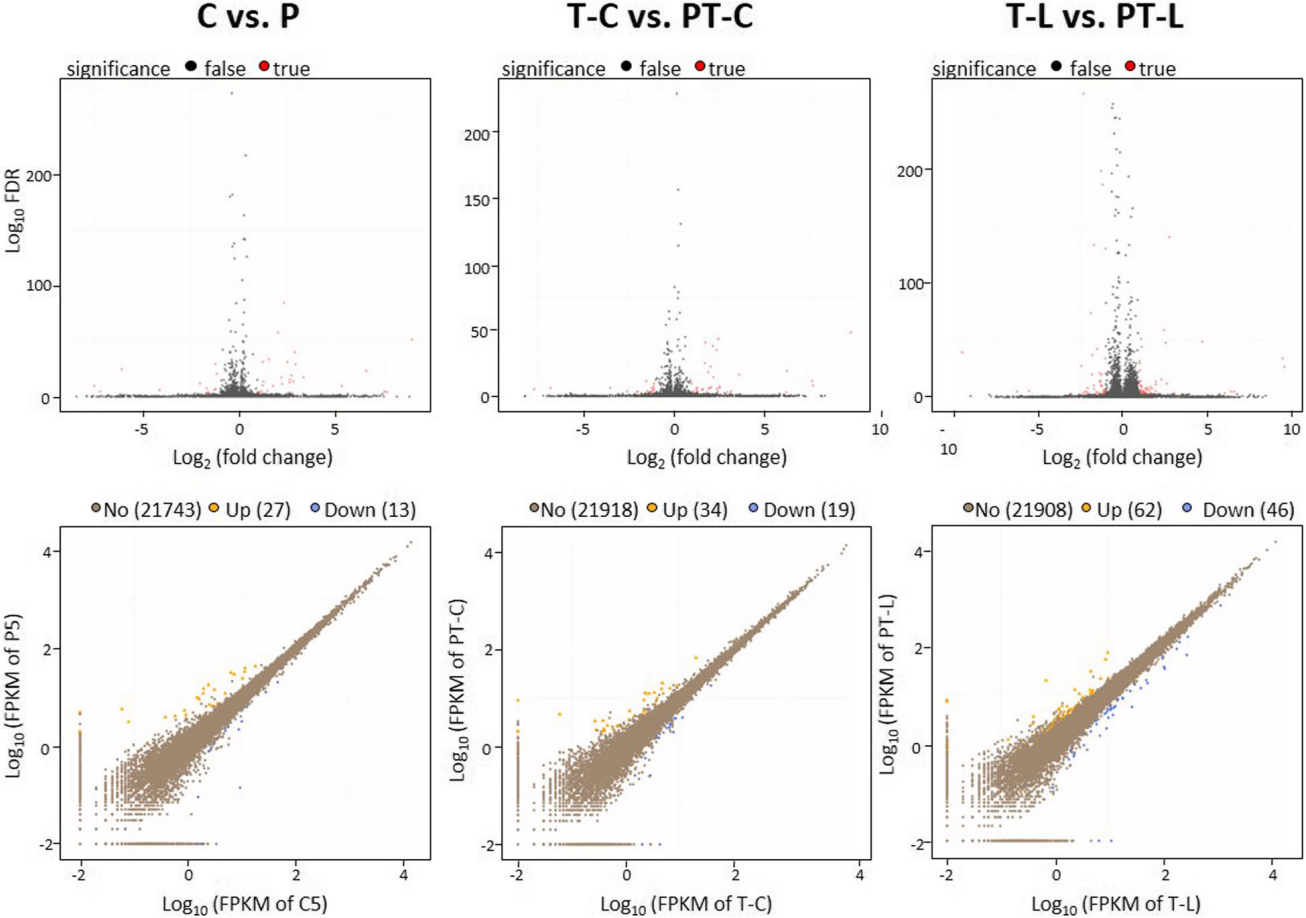
Statistical evaluation of priming-dependent regulation as obtained by RNASeq. Top: Volcano plots depicting genes with statistically significant regulation in red. Bottom: Comparison of the regulation intensity in primed (y-axis) and non-primed plants (x-axes). Genes with an FPKM value > 0.001 and up-regulated at least with log_2_ (primed / unprimed) = Ι 1 I are labelled in yellow, down-regulated genes in blue. Data for non-triggered plants are shown on the left, for cold-triggered ones in the middle panels and for light-triggered plants to the right

Volcano plots (depicting the intensity of priming-dependent regulation based on the false discovery rate (FDR)) (Fig. 4 top) and blotting of the gene expression levels of primed plants (y-axes) against the gene expression levels of the respective unprimed plants (x-axes) (Fig. 4 bottom), showed that cold priming affected cold and light regulation of only specific genes. Cold triggering resulted in much less gene expression variability than light triggering in cold-primed plants (Fig. 4 bottom). In general, most significant priming-dependent regulation was observed for medium strongly expressed genes (Fig. 4 bottom).

**Fig. 5:**
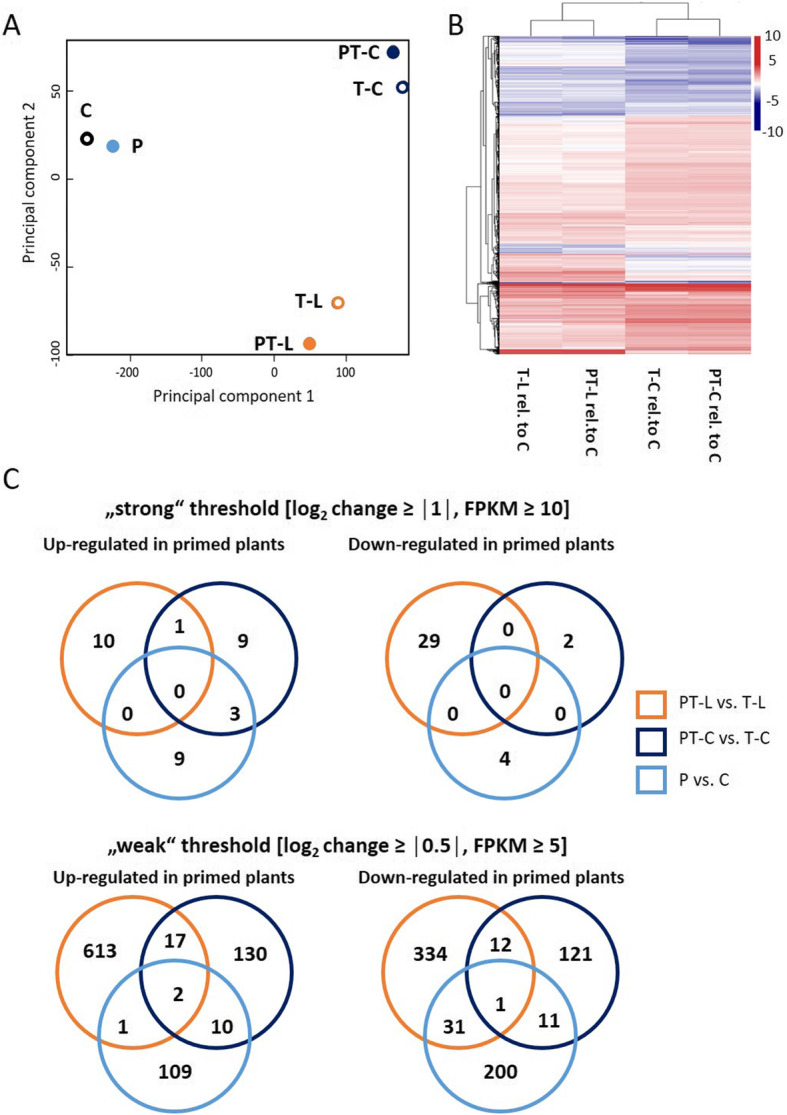
Transcript abundance regulation as observed by RNASeq. **a** Principal component analysis separating the data sets of non-triggered (C, P), cold-triggered (T-C and PT-C) and light-triggered samples (T-L and PT-L) stronger according to the type of the triggering stimulus than to the priming effect (P or PT in comparison to C or T). **b** Cluster analysis of transcript abundance regulation in cold- or light-triggered, primed or unprimed samples relative to the transcript level in control plants. The heat map lists only genes that were at least 2-fold stronger or less expressed in primed and / or triggered plants than in C-plants (FDR value < 0.001). 10-fold up-regulated transcripts are shown in dark red, 10-fold down-regulated transcripts are shown in blue. **c** VENN-diagrams depicting the number of genes up- or down-regulated in a priming-dependent manner before (P) and after cold (PT-C) or light triggering (PT-L) at a strong threshold setting of log_2_ (primed/unprimed) ≥ I 1 I and FPKM ≥10 (**top**) or a weak threshold setting of log_2_ (primed/unprimed) ≥ I 0.5 I and FPKM ≥5 (**bottom**)

Principal component analysis (PCA) (Fig. 5a) and clustering (Fig. 5b) of the relative transcript level in T- and PT-plants indicated that the priming effects on non-triggered, and cold- or light-triggered plants differed in direction and intensity. Already this first comparison let assume that the priming effects observed after triggering did not result from prolonged gene dysregulation in response to the priming stimulus, but that priming affected the response to the triggering stimulus.

### Long-term, not triggering-dependent gene expression effects of cold priming

For more stringent gene regulation analysis, the 13,775 genes were selected that were detected in all samples and were recorded with FPKM (fragments per kilobase of exon per million reads mapped) values of 5 or higher in at least one data set. The effects of priming on the transcript levels were calculated by dividing the FPKM-values of primed and non-primed plants at the end of the lag-phase (P / C) and in cold-triggered (PT-C / T-C) and light-triggered plants (PT-L / T-L).

Transcriptome comparison between C and P plants at the end of the 5-day-long lag-phase demonstrated that the transcriptome had widely reverted prior to application of the triggering stimuli. Only for 12 genes more than 2-fold higher and only for 4 genes more than 2-fold lower transcript levels were recorded in primed plants as compared to control plants (Fig. 5c top, Suppl. Tab. 3). At1g53870 (encoding a LURP (Late/sustained Up-regulation in Response to *Hyaloperonospora parasitica)*-like protein, At1g73260 (putative trypsin inhibitor), At4g12490 and At4g12480 (two bifunctional inhibitor proteins, AZI3 and EARLI1), a cation exchanger (At3g51860) and a haloacid dehalogenase-like hydrolase (HAD) superfamily protein (At5g36790) were strongest up-regulated. These genes were only weakly expressed under control conditions. Consequently, the absolute regulation of the transcript levels was low. On the contrary, the transcript levels of a transmembrane protein (At4g12495), the senescence and stress inducible gene *SAG13* (At2g29350, encoding a short-chain alcohol dehydrogenase) and *extensin-4* (At1g76930) were recorded with FPKM values higher than 10. Their transcript levels were more than 2-fold increased 5 days after cold priming reflecting a strong absolute effect (Supp. Tab. 3).

The four genes which were down-regulated in P compared to C encode lipid-transfer protein-4 (At5g59310), a glycine-rich protein (At1g04800), another LURP1-like protein (At1g53890) and an embryo development controlling gene (At4g29660) (Suppl. Tab. 3).

Analysing the transcript abundance patterns at lower threshold (FPKM ≥5 in at least one of the treatments and log_2_ (primed / unprimed) ≥ I0.5I) (Fig. 5c bottom) showed only for two of the 365 potentially long-term regulated genes, namely a hypothetical gene (At5g23411) and At1g53870 (encoding a LURP1-related protein), co-upregulation in not triggered and cold- or light-triggered plants. Only one hypothetical gene (At1g13470) was co-downregulated in cold-primed plants in all three treatment groups (Suppl. Tab. 4). The very low number of co-regulated genes demonstrates that the priming memory affects gene regulation in a stressor-specific manner.

### Common triggering-dependent effects of cold priming on cold and light triggering

Since cold and excess light regulate the majority of genes in the same direction [31], regulation of common signal transduction elements would result in high similarity between the effect of cold and light triggering on priming sensitive genes. Already the analysis of a small selection of *ZAT10*-related genes showed differences (Figs. 1b and 2e). On the transcriptome level, RNASeq analysis identified under the more stringent conditions used for analysis (FPKM ≥10 and log_2_(PT/T) ≥ I1I) only a gene for a not further characterized transmembrane protein (At4g22510) as potentially (at least 2-fold) priming co-regulated in cold- and light-triggered plants (Fig. 5c top).

Lowering the thresholds to FPKM ≥5 and log_2_(PT/T) ≥ I0.5I showed 29 genes as being co-regulated in a priming-dependent manner after light and cold triggering (Suppl. Tab. 4). Eight of the 17 co-up-regulated transcripts map to the same chromosome region and several of the short genes overlap in sense and antisense orientation. Consequently, the FPKM values (as calculated for these genes) may overstate the actual transcript abundances and the regulation amplitudes of individual genes. The remaining co-up-regulated genes encode (besides hypothetical proteins and proteins of unknown function) with ERD6-like 1 (*early response to dehydration-6 like-1*; At1g08920), a CC-NBS-LRR class immune receptor (At1g59218), the extensin OLE1 (At2g16630), a kinase inhibitor-like protein (At2g28870), plastome-encoded photoreceptor protein M (Atcg00220) and the plastid ribosomal subunit L32 (Atcg01020) a diverse spectrum of proteins.

In the group of the 12 genes, which are less expressed after light and cold triggering in primed plants (Suppl. Tab. 4), three encode disease associated genes, namely two β-glucanases (PR2 (BGL2; At3g57260) and BLG3 (At3g57240)) and one chitinase (At2g43570).

### Specific effects of cold priming on cold and light triggering

Most priming-responsive genes were regulated by either cold or by light triggering (Fig. 5c). Under highly selective conditions (FPKM ≥10 and log_2_(PT/T) ≥ I1I), the transcript levels of only two genes, expansin-A8 (At2g40610) and glycine-rich protein 9 (At2g05440), were lower after cold triggering due to cold priming. In parallel, 13 genes were more strongly expressed after cold triggering in cold-primed plants than in non-primed ones. Three of them, *Kunitz trypsin inhibitor 1* (At1g73260), *NIT2* (At3g44300) and *SAG13* (At2g29350), were already induced prior to application of the triggering stimulus. Nine of the remaining 10 genes encode (hypothetical) lipid transfer proteins or are not characterized for their function (Suppl. Tab. 3). The remaining, trigger-specifically regulated gene was *OLE1* (At2g16630) that encodes an extensin.

On the contrary, light triggering resulted in cold-primed plants in specific accumulation of the transcripts for 9 genes, of which three encode heat shock proteins. Various defence-related genes, such as *PR2* (pathogen responsive gene 2, At3g57260), *PR4* (At3g04720), a pathogen and circadian controlled gene *PCC1* (At3g22231), a chitinase (At3g12500) and five defensins, were less strongly induced by high light in primed plants than in naïve ones (Suppl. Tab. 3). Two genes, namely, At2g73260 and At4g12495, encoding a trypsin inhibitor and a transmembrane protein, showed inverse regulation in primed plants before and after light triggering. Inversion of the priming effect by the triggering response demonstrates that priming actively affected gene regulation by the triggering light stress event.

The quantitative differences between the priming impact on cold and light triggering were confirmed when the genes were filtered based on weaker criteria (FPKM ≥5 and log_2_(PT/T) ≥ I0.5I) (Fig. 5c): 130 genes were specifically induced and 121 down-regulated in cold-primed plants after cold triggering. Light triggering of cold-primed plants resulted in stronger induction of 613 and down-regulation of 334 genes in comparison to light-triggered non-primed plants.

### Analysis of regulation patterns by qPCR

**Fig. 6:**
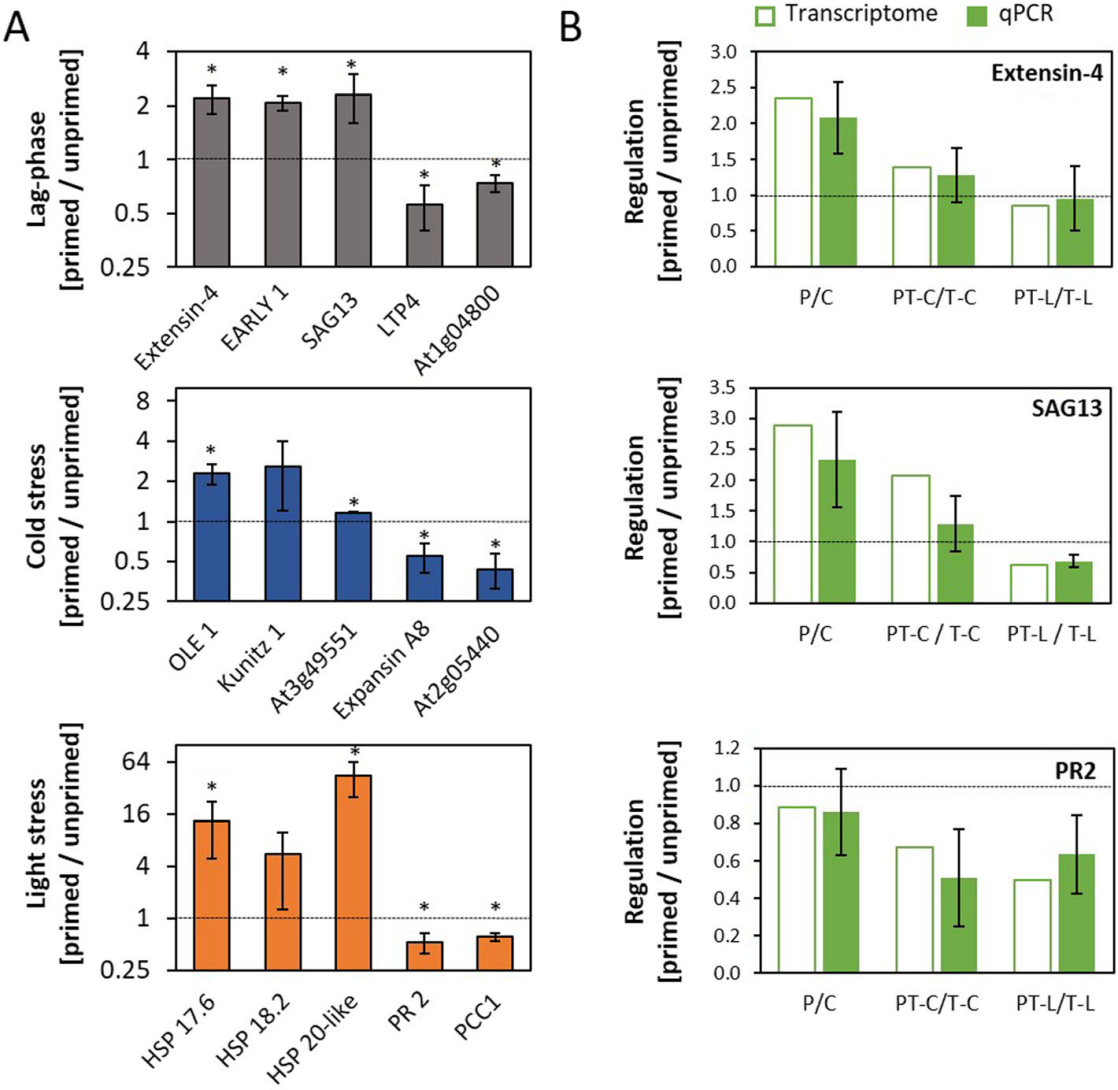
qPCR analysis of transcript abundance regulation. **a**: Consistency test on the regulation for genes showing strong regulation at the end of the lag-phase (top), after cold triggering (middle) or after light triggering (bottom). The transcript levels were quantified with gene specific primers and standardized on the transcript level of YLS8 in 3–5 independently cultivated and treated biological replicates. The figure shows means ± standard deviation. Statistical significance of regulation (Student t-test; p < 0.05) is labelled with an asterisk. **b**: Testing for consistency of regulation of the RNASeq analysis throughout the experiment for three selected genes. For all samples, the transcript abundance ratio between primed and unprimed plants obtained by qPCR in four independently cultivated and treated biological replicates (green bars; means ± standard deviation) was in the range of the ratio calculated based on the FPKM-values of the RNASeq analysis (white bars)

Regulation observed in the RNASeq experiment with pooled plant material from ten plants per treatment was evaluated by qPCR in at least 3 independently cultivated and treated biological replicates for 5 genes showing priming effects at the end of the lag-phase, for 5 genes which were regulated in a priming-dependent manner after cold triggering, and for 5 priming sensitive genes regulated by light (Fig. 6a). The priority was given to genes with high FPKM values. In the qPCR analysis, the transcript levels were normalized to the expression intensity of the constitutively expressed gene *YLS8* (At5g08290) [62]. In all three gene sets, three genes were selected which are up-regulated in primed plants as compared to non-primed plants and two which were down-regulated. 13, out of the selected 15 genes, showed in the qPCR analysis significant regulation (Student t-Test, *p* < 0.05) consistent with the RNASeq data. The transcript levels of the other two genes, namely At5g59720 (encoding the heat-shock protein HSP18.2) and At1g73260 (encoding a Kunitz factor protein) were by average (although not significantly) more than 2-fold regulated in the same direction as in the RNASeq experiment.

Of the five genes tested by qPCR for higher transcript levels 5 days after cold priming (Fig. 6a top), RNASeq analysis indicated only for *SAG13* also higher transcript levels after cold triggering. qPCR in independently cultivated and treated biological replicates confirmed this effect (Fig. 6b). Additionally, it also showed down-regulation in primed plants after light triggering consistent with the RNASeq analysis (Suppl. Tab. 3; Fig. 6b). qPCR further confirmed the regulation observed by RNASeq for extensin-4 (At1g76930) and *PR2* (At3g57260) before and after triggering (Fig. 6b). The ratios calculated from the FPKM values of primed and the respective unprimed plants (P/C; PT-C/T-C and PT-L/T-L) were for all treatments in the range of the values obtained by qPCR for the various biological replicates (Fig. 6b).

### Functional categorization of the cold priming effect on the triggering response

**Fig. 7:**
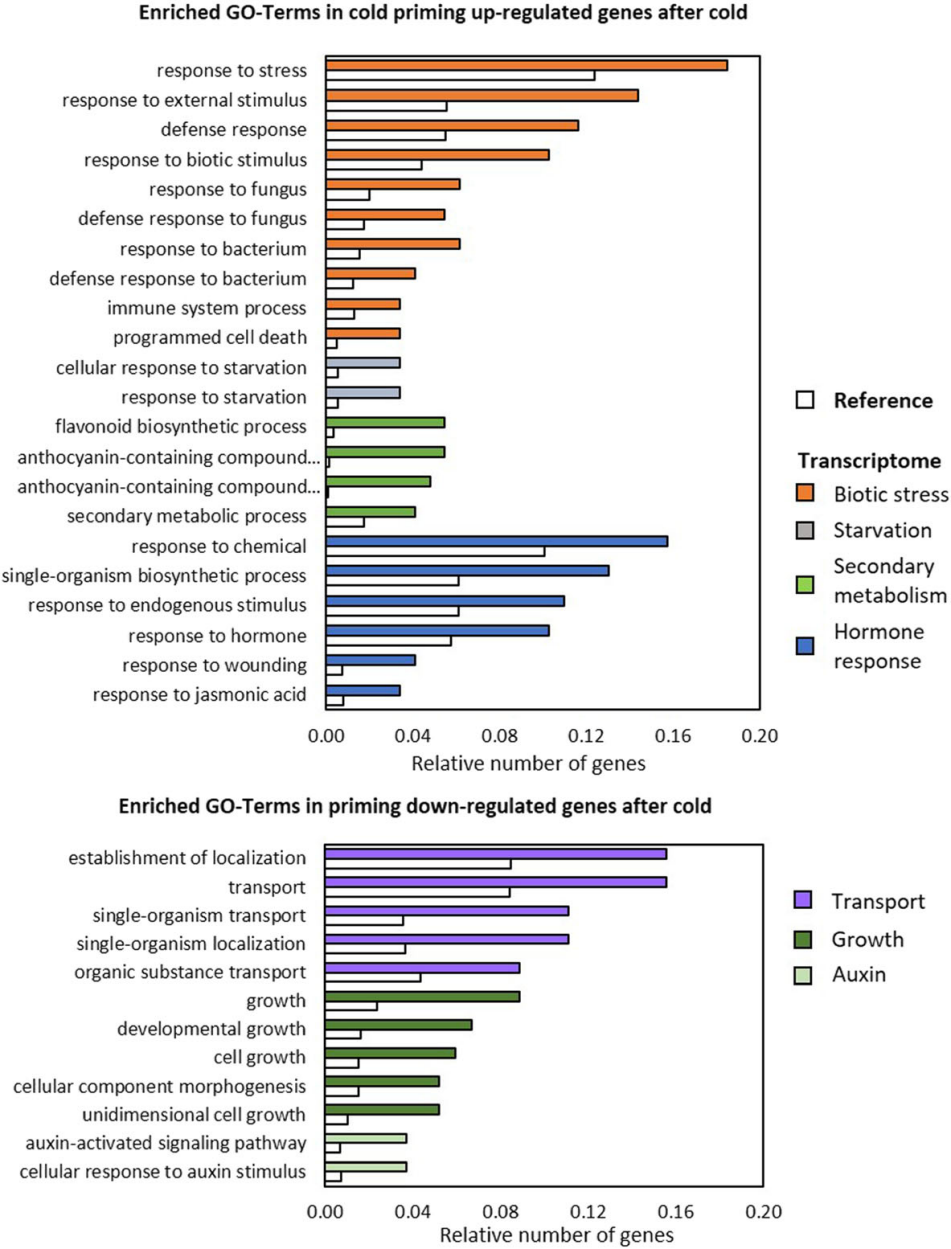
Functional characterization of genes regulated in a priming-dependent manner after cold triggering. Enriched functional gene ontologies were identified with AgriGO using TAIR10 as background. The crude data including statistical information and the GO-term reference codes are summarized in Suppl. Tab. 5

**Fig. 8:**
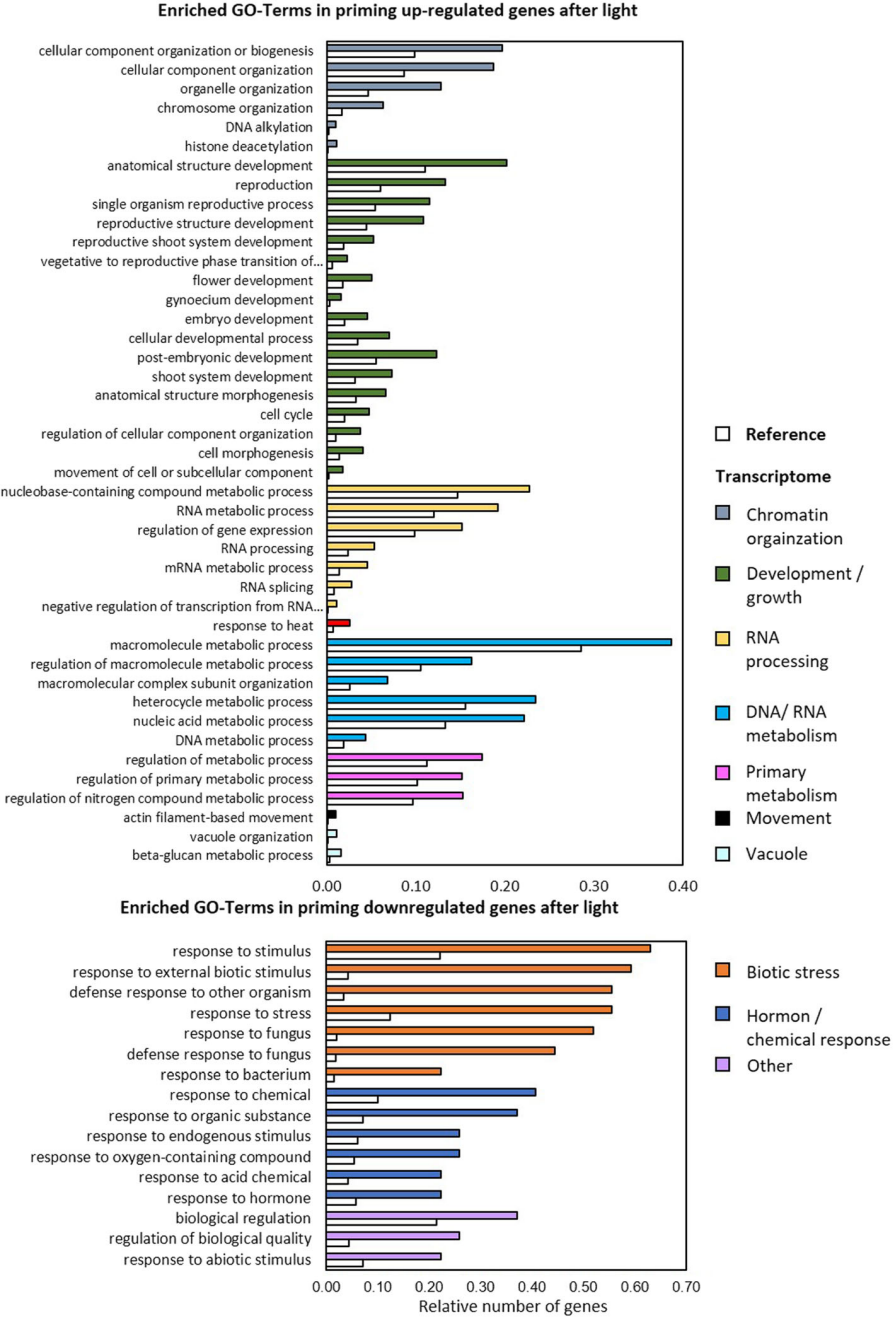
Functional characterization of genes regulated in a priming-dependent manner after light triggering. Enriched functional gene ontologies were identified with AgriGO using TAIR10 as background. The crude data including statistical information and the GO-term reference codes are summarized in Suppl. Tab. 5

Functional categorization of the priming-regulated genes based on analysing the enrichment of gene ontologies (GO) [63, 64] was performed with the wider data set (log_2_ (PT/T) > l0.5l FPKM ≥5) on the AgriGO v2 platform (http://systemsbiology.cau.edu.cn/agriGOv2/). Data processing was evaluated using the Fischer test (F-test) and the Yekutieli method for α-level adjustment at a p-level of 0.05 [65]. The minimum threshold for statistical testing and multi-test adjustment was set to 5 genes per GO-term [66]. From the primary data, the subset of the most specific GOs within the hierarchical GO structure were extracted for the figures (Figs. 7 and 8). The full lists including information on the p-value and FDR (False Discovery Rate) and graphical images depicting all GO-terms in hierarchical order are provided in the supplements (Suppl. Tab. 5).

In the group of transcripts that were up-regulated in cold-primed plants after cold triggering, stress regulated genes were significantly enriched in comparison to non-primed cold stressed plants (Fig. 7). Especially genes responding to wounding, immune and programmed cell death regulation and / or genes under control of jasmonic acid signalling were over-represented. Additionally, priming preferentially affected the cold triggering response of genes involved in the starvation regulation and in flavonoid and anthocyanin biosynthesis (At4g22880, At4g09820, At2g02990, At3g29590, At5g17220, At5g42800, At4g14090, At5g54060). All eight genes of the latter group were also induced by excess light but were less induced or even inversely regulated in primed plants after light triggering compared to primed plants after cold triggering (Suppl. Tab. 5). *CHS* and *PAL1*, which regulate early steps of phenylpropanoid metabolism and were previously shown to be more strongly activated in cold-primed plants upon cold triggering [17], were also more strongly induced in cold-primed plants in response to cold triggering in the new dataset, although they did not pass the threshold criteria used here for the bioinformatics analysis. In parallel, cold triggering resulted in cold-primed plants in weaker expression of genes involved in transport organization, growth and morphogenesis (Fig. 7). Various of the less expressed genes respond to auxin-activated signalling and response pathways.

After light triggering, genes involved in organelle organization, morphogenesis and nucleic acid metabolism were more strongly induced in cold-primed plants than in non-primed ones. Genes responding to biotic stimuli, acids and oxygen-containing organic compounds (At5g44420, At3g15356, At3g22231, At2g14560, At1g73260, At4g10500, At3g16530) and genes involved in metabolic regulatory processes are less represented in primed plants (Fig. 8). In general, GO analysis showed that cold priming results in an inverse support of growth and biotic stress response upon cold and light triggering (Figs. 7 and 8, orange and dark green bars).

### Sub-analysis of the priming-responsive genes inversely regulated by cold and high light

In the group of 159 genes with higher transcript levels in PT-C (cold-primed and cold-triggered) plants than in T-C (only cold-triggered) plants and the 379 genes down-regulated in PT-L (cold-primed and light-triggered) plants as compared to T-L (only light-triggered) plants (FPKM values > 5 and log_2_ (PT/T) > l0.5l) 17 genes were inversely regulated (Supp. Tab. 6). Additionally, 12 genes were inversely regulated between the group of 145 genes down-regulated PT-C plants (as compared to T-C) and 633 genes up-regulated in PT-L (as compared to T-L) (Supp. Tab. 6).

Six of these (in total) 29 inversely regulated genes were not annotated in TAIR10, which is the data background used for functional categorization with AgriGO v2. Only one biological function was significantly overrepresented in the remaining group of 23 genes (Suppl. Tab. 7). Seven of the 23 genes, namely At2g29350, At4g37990, At1g73260, At2g43510, At3g22231, At3g04720 and At3g12500, respond to biotic stimuli. They all showed higher transcript levels after cold triggering and lower ones after light triggering if the plants were cold-primed before (Suppl. Tab. 6). Taking even slight regulation prior to triggering into account, all these genes show specific responses to light triggering (Suppl. Tab. 6). Three of them (At3g22231, At3g04720 and At3g12500) showed also up-regulation of the transcript levels after cold triggering and down-regulation in response to light. These three two-directionally regulated genes encode the plasma membrane protein *Pathogen and Circadian Controlled 1* (PCC1; At3g22231), *Pathogenesis Related 4* (PR4; At3g04720) and a basic chitinase (CHI-B; At3g12500). All three genes are associated with pathogen defence. Also *CHS (At5g13930)*, but not the other core response gene *PAL1 (At2g37040),* showed stronger expression in cold-primed plants upon cold triggering and lower transcript levels after excess light triggering, although with lower amplitudes than *PCC1*, *PR4* and *CHI-B* (Suppl. Tab. 2).

**Fig. 9:**
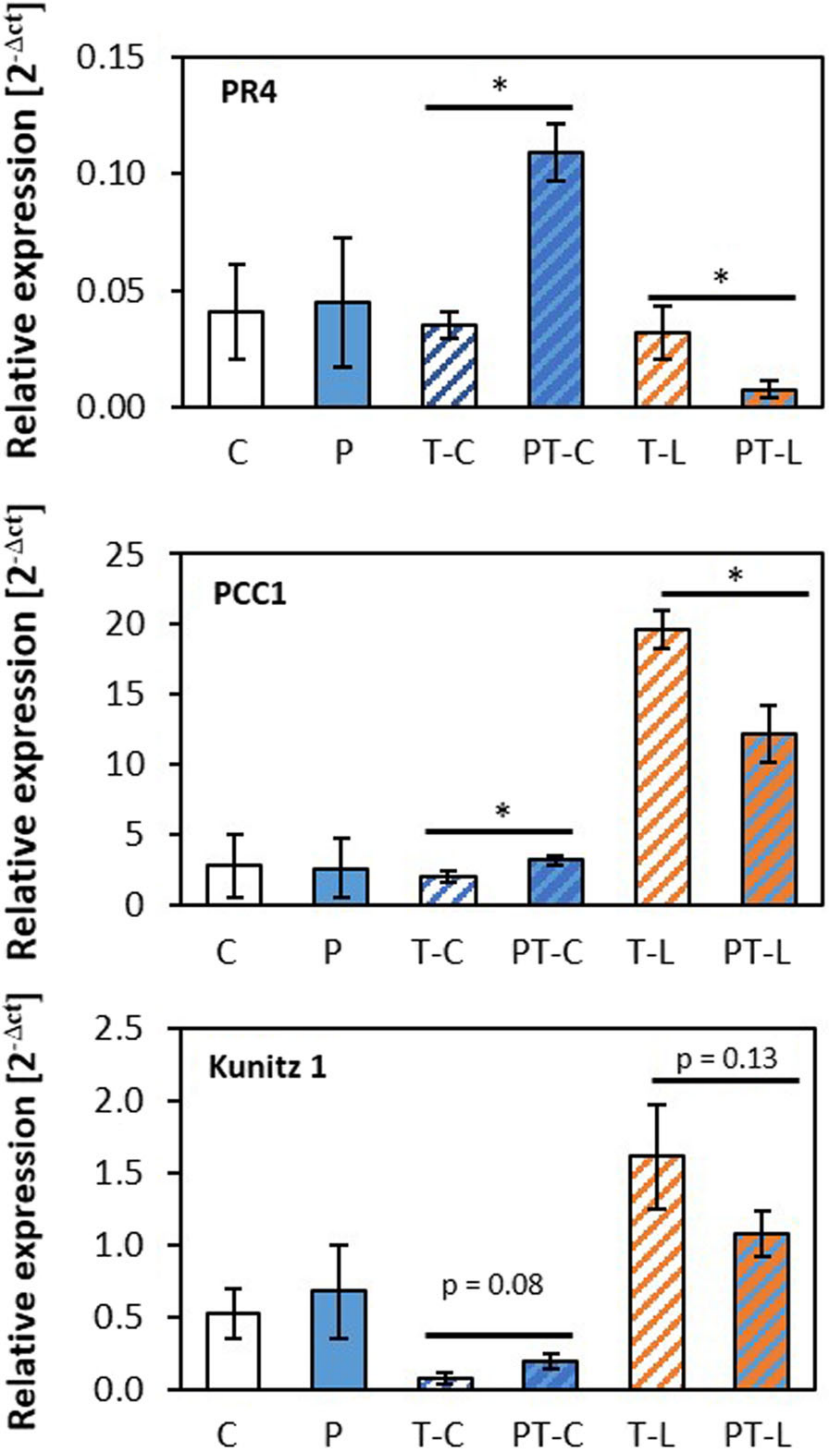
qPCR confirmation of inverse regulation of three pathogenesis-associated genes. The transcript levels were quantified with gene specific primers and standardized on the transcript level of YLS8 in 3 independently cultivated and treated biological replicates. The figure shows means ± standard deviation. Statistical significance of regulation (Student t-test; *p* < 0.05) is labelled with an asterisk

Expression network analysis on the GENEMANIA platform indicated only very faint co-expression between *PR4* and *CHI-B* and no co-regulation of the two genes with *PCC1*. The impression that these genes are hardly co-regulated in naïve plants was confirmed by comparison of transcript abundance regulation using the compare-mode of the eFP browser [67] on publicly available transcript abundance regulation data for developmental regulation in *Arabidopsis thaliana* and the response to biotic and abiotic stress. qPCR analysis confirmed the inverse regulation of pathogen related genes *PCC1*, *PR4* and *Kunitz 1* after cold and light triggering of cold-primed plants (Fig. 9). For CHI-B, the transcript levels were below the detection level of qPCR.

The other 16 genes, which responded inversely to cold and light triggering in a priming-dependent manner, have diverse functions. Five encode transmembrane proteins (At4g12495, At1g79170, At1g16916, At5g65580 and At1g53035), two protease inhibitors (At1g73260 and At2g43510) and two protein phosphatases 2C (At5g02760 and At3g16800) (Suppl. Tab. 6).

## Discussion

Stresses activate a sequence of events which start within seconds to minutes with the first measurable changes in transcriptional activity [68, 69]. After a period of massive regulation, the transcriptome gets adjusted to regulation of acclimation. Inactivation of primary stress regulation and secondary regulation dominate the post-stress phase [70]. In our experiment, 5 days after the plants perceived the priming cold stimulus, primary and secondary gene expression regulation was almost fully reset (Supp. Tab. 2; Fig. 5). At this stage, we exposed the plants either to cold or to excess light. The two stresses, if applied to naïve plants, regulate the majority of genes in the same direction [18, 31] (Suppl. Tab. 2). 5 days after cold priming, however, the same stimuli caused mainly specific effects and partly even inverse transcript abundance regulation (Figs. 1, 2, 5 and 6; Suppl. Tab. 2, 3 and 4). Due to the low overlap between genes that were cold and light regulated in a priming-dependent manner (Figs. 1, 2 and 5 plus Suppl. Tab. 3, 4, 5 and 6), we conclude that cold priming uncouples cold and light regulation of specific genes.

The mechanisms, by which cold priming establishes the memory and how the priming-induced information is recorded in primed plants, are still under investigation [13, 15, 16]. Various studies suggest an epigenetic memory, such as histone and DNA (de-)acetylation or (de-)methylation, for storing information on thermal stress events [15, 71–73]. For example, *COR15A* (At2g42540) and *COR47* (At1g20440) are more strongly expressed, if the second cold stimulus quickly follows the first one [74]. The majority of the cold-induced histone marks, however, is metastable. Consequently, the effects on gene expression regulation get quickly lost. For example, the cold-priming effect on *COR15A* can fully revert within 24 h, if priming was performed with a short cold stimulus [72]. On the contrary, prolonged cold, such as 2 weeks at 4 °C, leads to higher transcript accumulation of *COR15A* upon a 5 day later applied 24 h 4 °C triggering stimulus [17]. Transformation of a metastable cold memory into a stable one, such as in the regulation of *FLOWERING LOCUS C* (*FLC*; At5g10140), requires several days or even several weeks of cold exposure [75]. Consistent with the previous qPCR-based analysis of *COR15A* regulation [17], 5 days after 24 h cold priming none of the reference genes for epigenetic cold memories, namely *COR15A*, *COR47* or *FLC* (Suppl. Tab. 2) [74, 75], showed priming-dependent regulation in our present study.

Despite widely overlapping light and cold effects on the transcriptome of naïve plants [31] (Suppl. Tab. 2), priming-dependent co-regulation was only observed for 32 genes (of which several overlap and might reflect double annotations) (Fig. 5b). 29 priming-sensitive genes were even inversely regulated. The latter group included well characterized genes of the core environmental stress response cluster [18], such as ZAT transcription factors, *CHS* and the pathogenesis associated genes *PCC1*, *PR4* and *CHI-B* (Suppl. Tab. 2; Fig. 9). Cold-priming supported expression of stress (hormone) responsive genes upon cold triggering and resulted in lower expression of genes related to growth and metabolite transport (Fig. 7). Genes with functions in stress response regulation were down-regulated in cold-primed plants after light triggering and genes involved in growth, metabolism and development were up-regulated (Fig. 8). Such inverse effects of cold priming on gene expression regulation demonstrated that priming affects cold- and light sensitivity and responsiveness in a stressor specific manner. With respect to biological function, our analysis highlighted two cold-priming effects:

1. *Cold priming supports cold regulation of genes involved in anthocyanin and flavonoid metabolism.*

Eight genes involved in the biosynthesis of flavonoids and anthocyanins showed priming dependent regulation in response to cold triggering (Fig. 7; Suppl. Tab. 2 and 5). Anthocyanins and flavonoids are broad spectrum protectants that not only filter ultra violet (UV) - and / or blue and red light, but have also antioxidant capacities [76, 77]. Their synthesis is activated by various stresses, including cold, UV light, drought, salt and high light [78, 79]. *CHS* and *PAL1*, which were previously shown by qPCR to be more strongly induced in cold-primed plants upon cold triggering [17], encode enzymes catalysing initial steps of phenylpropanoid metabolism and controlling the flux capacities into chalcone metabolism. Although regulation of *CHS* and *PAL1* transcript levels did not pass the strict threshold criteria applied in this study, their transcript abundances were also higher in cold-primed plants upon cold triggering (and lower or unchanged upon high light triggering) (Suppl. Tab. 2). Five of the eight cold-regulated genes, namely At5g42800, At5g17220, At4g22880, At4g14090 and At4g09820, plus *CHS* and *PAL1* are activated by MYB75 (At1g56650) [80]. *MYB75* expression is regulated by ZAT10 [20]. *MYB75* transcript levels showed slight positive cold priming effects upon cold triggering, but not upon high light triggering (Suppl. Tab. 2), consistent with selective priming-dependent regulation of *ZAT10* upon cold, but not light triggering (Figs. 1 and 2). It could link priming regulation of *CHS* / *PAL1* and *ZAT10*, which we previously hypothesized to be controlled by parallel induced, inversely acting pathways [17].

(2) *Inverse cold-priming dependent regulation upon cold and light triggering.*

The most striking observation of our study was the inverse trade-off between the support of growth and defence upon cold and light triggering after cold priming. Cold pre-treatment is well known to decrease plant susceptibility to pathogens [81]. A recent transcriptome analysis, showing lower susceptibility of cold pretreated Arabidopsis against the pathogenic bacterium *Pseudomonas syringae* (*Pst*) strain DC 3000, explained the effect by cold-modulation of salicylic acid biosynthesis and signalling [82]. Salicylic acid levels, that increase in the cold [83], can activate local as well as systemic resistance [84]. Only 2 h after the 10 h long cold-priming treatment, cold-modification of defence signalling resulted in stronger expression of *PAL1* and *PR2*, and weaker induction of *PR4* upon infiltration with *Pseudomonas syringae DC3000* [82]. In our study, 5 days after 24 h cold priming, *PAL1* was more strongly induced by cold and by high light in cold-primed plants. However, the transcript levels of the more specific salicylic acid regulated gene *PR2* were lower (Fig. 6 Suppl. Tab. 2) after both stress treatments if the plants were cold-primed. On the contrary, the gene for the chitin binding protein *PR4* and *PCC1* were not down-regulated, but strongly up-regulated by cold, and down-regulated by light in cold-primed plants (Fig. 9). In our opinion, such specific regulation of defence related genes upon cold- and high light triggering can, like differential regulation of *ZAT10* (Figs. 1 and 2), only be explained by specific modulation of the gene responsiveness to the specific trigger. In other words, we conclude that the cold priming memory uncouples core stress signalling and deploys its regulatory potential on stressor specific gene regulation.

## Conclusions

Controlling the balance between defence and growth is crucial for plants in a changing environment in order to optimize their fitness [85]. Our study demonstrated that cold priming differentially modifies regulation of specific genes and even uncouples regulation of genes of the core environmental response cluster. Transcriptome wide analysis of the consequences of cold priming demonstrated that cold triggering supports expression of various genes involved in defence and protection on the cost of the expression of transport and growth-related genes. On the contrary, light triggering preferentially activates genes involved in metabolism and development, but down-regulates genes involved in the defence response. The overall pattern is manifested in the inverse regulation of 29 genes. From this, we conclude that cold priming modifies stress signalling by differentiating cold and light induced regulation.

## Methods

### Plant growth and stress treatments

**Fig. 10:**
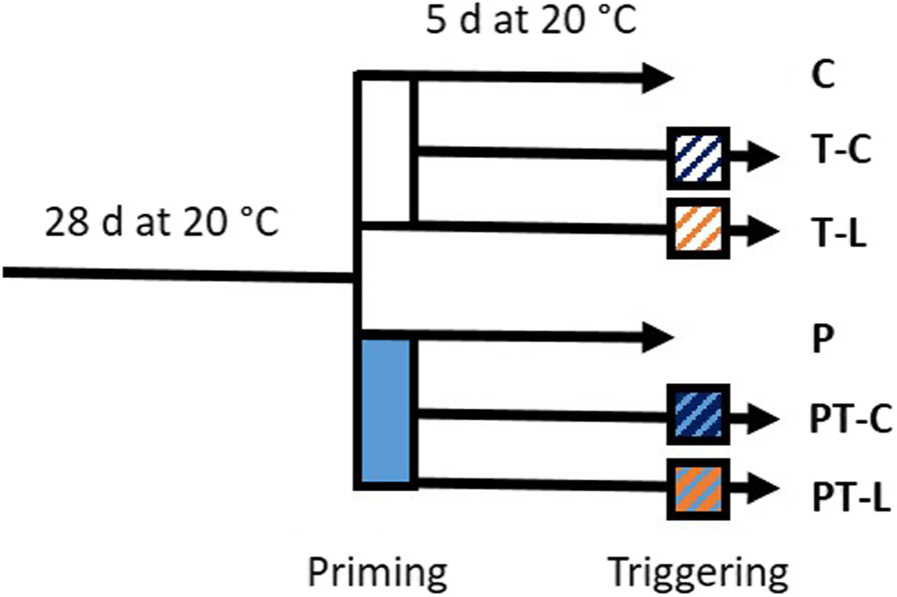
Experimental set-up. At an age of 28 days half of the plants were cold treated for 24 h at 4 °C (priming) and then retransferred to 20 °C and an illumination intensity of 100–110 μmol quanta m^− 2^ s^− 1^. 5 days later 1/3 of the plants of each set was cold-triggered for 24 h at 4 °C (T-C and PT-C), 1/3 light-triggered for 2 h at 800 μmol quanta m^− 2^ s^− 1^ (T-L and PT-L). The remaining plants (control plants: C; only primed plants: P)) were harvested at the end of the lag-phase

*Arabidopsis thaliana* (Col-0) plants were grown for 28 days individually in round pots (6 cm diameter) in soil at 20 ± 2 °C at a day - night regime of 10 h light / 14 h dark and an illumination rate of 100–110 μmol quanta*m^− 2^ s^− 1^ (Lumilux Cool White fluorescence stripes; Osram, Germany). For priming, a 24 h cold stimulus was imposed to half of the 4-week-old plants by transferring them 2.5 h after the onset of light to a 4 ± 2 °C cold chamber with the same aeration, illumination and air humidity setting as the 20 °C chamber (Fig. 10). Afterwards the primed plants were placed back to 20 °C and further cultivated side-by-side with non-primed plants in a randomized pattern. The general settings were identical to those used in the previous study [17], except that the temperature sensor in the cold chamber was exchanged to one shortening the phase length in the cooling rhythm, which better stabilizes the day and night temperatures. 1/3 of the primed and naïve plants was harvested 5 days after the end of the priming stimulus 2.5 h after onset of light. The control plants (C plants) were kept all time at 20 °C.

Cold triggering was started after a lag-phase of 5 days at 20 °C with 1/3 of the primed and 1/3 of the control plants by transfer of the plants to 4 °C (cold triggering) (Fig. 10). For high light triggering, 1/3 of the primed and 1/3 of the naïve plants were exposed for 2 h to a photon flux density of 800 μmol quanta m^− 2^ s^− 1^ 30 min after the onset of light using halogen lamps (R7-s 500 W, Emil Lux GmbH Wermelskirchen, Germany). The heat emission of the halogen lamps was filtered through a water layer and additionally controlled by moderate ventilation. The leaf temperature was monitored on the upper leaf surface with an infrared thermometer.

For the RNASeq samples, entire rosettes of ten plants were harvested per treatment at the end of the 5-day-long lag-phase and 2 h after the cold and excess light triggering treatment, combined and immediately frozen in liquid N_2_ to stabilize the RNA against degradation. For each of at least 3 independent experimental replicates used for the qPCR analysis, entire rosettes of 5–7 individual plants per treatment and sample were harvested, pooled and also immediately frozen in liquid N_2_.

### RNA-isolation and RNA library construction

For RNA isolation, the plant material was ground to a fine powder in liquid N_2_. RNA was extracted from 100 mg plant material using the Gene Matrix Universal RNA Purification Kit (EURx, Gdansk, Poland) including the DNase treatment recommended by the supplier. The RNA was precipitated from the solution overnight at − 20 °C by adding 0.1 volume of 3 M sodium acetate (pH 5.2) and 2.5-volume absolute ethanol. After dissolving the RNA in 50 μl RNAse-free H_2_O, the RNA integrity was assessed by electrophoresis on a 2% (w/v) agarose gel supplemented with 1% (v/v) formaldehyde.

For RNA library construction, the mRNA was enriched using oligo (dT) magnetic beads and depleted from rRNA by DNA/rRNA hybridization according to standard procedures of the Beijing Genomics Institute (Beijing, China). Afterwards, the mRNA was transcribed into cDNA and the second DNA strand was generated with random N_6_ primer. The double stranded cDNA was then 5′-end repaired, 3′-poly-A-tailed and ligated with an oligo-dT-adapter. The ligation product was amplified with specific adapter primers by PCR. Single-end sequencing on the Illumina High-Seq4000 platform of the Bejing Genomics Institute led to an average of 24 million (± 160.000) reads with a read length of 50 base pairs per treatment (Suppl. Tab. 1).

### Bioinformatic analysis

The reads obtained by RNASeq, that did not contain adaptor sequence contaminations and less than 10% unclear bases (= clean reads), were aligned to the Arabidopsis reference genome (TAIR10) using Bowtie (version 2.1.0 [86];) and HISAT (2.1.0 [87];). The number of aligned reads were normalized for each gene to the transcript length and the total number of reads per treatment by using the RSEM software package. For the 2000 highest expressed genes a principal component analysis was performed by using edgeR [88] and limma libraries [89] in R 3.5.1. Correlation analysis of differentially expressed genes for the heat map was performed by using the R function pheatmap (https://CRAN.R-project.org/package=pheatmap). Gene co-regulation was analysed with InteractiVenn [90]. Additional expression analyses were performed with GENEMANIA [35], STRING v.11 [36] and on the eFP browser platform [67]. The functional characterization by gene ontologies of significantly expressed and differentially regulated genes (log_2_-change > 1 and a FPKM value > 5) was performed on the AgriGO 2.0 platform [66] according to gene ontology terms of the TAIR10 release.

### Reverse transcription and quantitative real time PCR

For the real-time PCR analysis, the mRNA was transcribed into cDNA using the High Capacity Reverse Transcription Kit (Applied Biosystems, Carlsbad, CA, U.S.A.). Oligo dT_16_V primer were annealed to the polyA-tails of the mRNA during 10 min incubation at 25 °C. After 2 h at 37 °C, the reaction was stopped by 5 min heat inactivation of the enzyme at 85 °C. Real-time qPCR analysis was performed on the CFX96 real-time system (Biorad, Hercules, CA, U.S.A.) with 50 ng template cDNA and 0.2 μl 10xSYBR Green (Sigma-Aldrich, Germany) in 20 μl as described previously [17], except that the transcript levels were only standardized on *YLS8* (*yellow leaf specific protein 8*; At5g08290), since other typically in qPCR analysis used reference genes responded either to the cold or to the light treatment. All primers, if applicable, were designed to span exon-intron borders by the QUANTPRIME software [91]. Primer sequences are listed in the supplements (Suppl. Tab. 8).

### Quantification of H_2_O_2_ levels

Medium old leaves, which show strongest priming sensitivity [30] and lowest background H_2_O_2_ levels [92], were frozen immediately in liquid nitrogen to fix metabolism. 50 mg of the frozen plant material was homogenized in 200 μl 5 mM KCN according to [93]. After sedimentation of the insoluble material (5 min at 13.000 g and 4 °C), 100 μl of the supernatant was mixed with 1 ml precooled dye solution (100 volumes 125 μM xylenol orange in 100 mM sorbitol freshly mixed with 1 volume of 25 mM (NH_4_)_2_Fe(SO_4_)_2_ in 2.5 M H_2_SO_4_). The absorbance was determined spectrophotometrically at 560 nm after 10 min incubation at room temperature and quantified based on a calibration curve obtained with H_2_O_2_ standards.

### Chlorophyll-a fluorescence analysis

After 20 min dark acclimatization, the maximal chlorophyll-a fluorescence (F_V_/F_M_) was determined in the medium old rosette leaves with a saturating light flash (> 1000 μmol quanta m^− 2^ s^− 1^) in primed (P) and primed + triggered (PT) plants in an Imaging PAM IMAG-K4B (Walz, Effeltrich, Germany). The effective quantum yield of photosystem II (Φ_PS-II_ = (F_M´_- F)/F_M´_)), photochemical quenching (qP = (F_M´_ - F)/(F_M´ -_ F_0´_)) and non-photochemical quenching (NPQ = (F_M_ /F_M´_) – 1) were analysed with saturating light flashes spaced by 20 s time gaps before and during illumination with a photosynthetic photon flux density of 185 μmol quanta m^− 2^ s^− 1^ in the light exposed upper third of the leaves.

### Statistical analyses

The significance of difference was evaluated with Student t-tests (*p* < 0.05) if two data sets were compared. Larger data sets were analysed with the Tukey posthoc test (*p* < 0.05) using the R 3.5.1 software package.

## Supplementary information

**Additional file 1:Suppl. Table 1.** General information on the quality of RNA sequencing and RNA sequence alignments.

**Additional file 2:Suppl**. **Table 2.** FPKM values in control plants (C5) and primed plants (P5) at the end of the 5 day long lag-phase, in only cold (T-C) or only light-triggered (T-L) plants and in cold-primed and cold (PT-C) or light-triggered (PT-L) plants arranged according to the FPKM-value in control plants. For each gene, the transcript variants used for aligning the RNASeq reads are listed.

**Additional file 3:Suppl**. **Table 3.** Identities of the genes showing priming-dependent regulation at a threshold of log_2_ (primed/unprimed) ≥ I 1 I and FPKM ≥10 in at least one treatment. log_2_ (primed/unprimed) values ≥1 are highlighted in orange, log_2_ (primed/unprimed) values ≤ − 1 in blue. Genes tested by qPCR for their regulation are highlighted in bright yellow.

**Additional file 4:Suppl**. **Table 4.** Identities of the genes showing priming-dependent regulation at a threshold of log_2_ (primed/unprimed) ≥ I 0.5 I and FPKM ≥5 in at least one treatment. log_2_ (primed/unprimed) values ≥0.5 are highlighted in orange, log_2_ (primed/unprimed) values ≤ − 0.5 in blue. Genes tested by qPCR for their regulation are highlighted in bright yellow.

**Additional file 5:Suppl**. **Table 5.** Crude data on the gene ontologies (GO-terms) in the functional enrichment analysis of genes regulated at a threshold of log_2_ (primed/unprimed) ≥ I 0.5 I and FPKM ≥5 after priming. The table lists (from left to right) the number of genes associated with the GO-term in the query, the number of genes in the query, the number of genes associated with the GO-term in the reference background (TAIR10), the total number of genes used as background, the ratio of genes associated with the respective GO-term relative to the total number of genes analysed in the query (transcriptome) and in the background (reference), the enrichment in the query relative to the reference, the p-value for the significance of enrichment and the false discovery rate (FDR).

**Additional file 6:Suppl**. **Table 6.** Identities of the genes showing inverse priming-dependent regulation after cold and light triggering. The seven genes associated with defence responses are labelled in dark orange. log_2_ (primed/unprimed) values ≥0.5 is highlighted in orange, log_2_ (primed/unprimed) values ≤ − 0.5 in blue.

**Additional file 7:Suppl**. **Table 7.** Gene ontologies (GO-term) obtained by functional enrichment analysis of genes which were inversely regulated by cold and light in a priming-dependent manner (Suppl. Tab. 6). The GOs grouping the seven genes mentioned in the text are labelled in orange.

**Additional file 8:Suppl**. **Table 8.** List of oligonucleotide primers used for the qPCR analysis.

## Data Availability

All the datasets generated and analysed during the current study were uploaded as with the manuscript as additional files. Primary and processed data are available in Suppl. Tab. *2*, on PrimeDB (https://primedb.mpimp-golm.mpg.de/index.html?sid=reviewer&pid=bf0fa52fb0b4e9641017a9d0b6528261) and on NCBI-GEO (GSE151749; GSM4589752 – GSM4589757).

## Abbreviations

C: Control plants
C5: Control plants at the end of the lag-phase
CBF: C-repeat binding factor
Col-0: *Arabidopsis thaliana* var. Columbia-0
FPKM: Fragments per kilobase of exon per million reads mapped
F_V_/F_M_: Quantum yield of photosystem II in dark acclimated plants
HSF: Heat-shock factor
ICE: Inducer of CBF expression
MAP: Mitogen activated protein
MPK: MAP protein kinase
P: Primed plants
P5: Primed plants at the end of the lag-phase
PPFD: Photosynthetic photon flux density
PT: Primed and triggered plants
PT-C: Cold-primed and cold-triggered plants
PT-L: Cold-primed and light-triggered plants
RNASeq: RNA-Sequencing
ROS: Reactive oxygen species
sAPX: Stromal ascorbate peroxidase
T: Plants which only faced the triggering stimulus
T-C: Cold-triggered plants
T-L: Light-triggered plants
tAPX: Thylakoid-bound ascorbate peroxidase
qP: Photosynthetic quench
qPCR: Real-time quantitative polymerase chain reaction
GO: Gene ontology
NPQ: Non photochemical quench
UV: Ultra violet
Φ_PS-II_: Quantum yield of photosystem II in the light
